# A study on the quality evaluation index system of smart home care for older adults in the community ——based on Delphi and AHP

**DOI:** 10.1186/s12889-023-15262-1

**Published:** 2023-03-01

**Authors:** Huaxiao Chen, Yuwei Zhang, Li Wang

**Affiliations:** Institute of Health Management, Southern Medical University, No.1023-1063 Shatai Road, Baiyun District, Guangzhou City, Guangdong Province China

**Keywords:** Community-dwelling older adults, Smart home care evaluation index, Service quality model, Delphi method, Analytic hierarchy process (AHP)

## Abstract

**Background:**

In the context of the “silver wave” and “technology wave”, smart home care for older adults in the community provide new ways for China and other countries to support ageing in place. Yet, only very few studies have focused on developing a quality index system of smart care. This study attempted to draw on the SERVQUAL model to establish a quality evaluation index system for smart senior care for older adults in the community.

**Methods:**

On the basis of the service quality model, this paper has integrated qualitative and quantitative analyses using the Delphi and Analytic Hierarchy Process (AHP) methods to construct the index system of smart home care in the community and obtain the weights. These were based on literature research and field interviews in Guangzhou and Shenzhen pilot districts.

**Results:**

A quality evaluation indexes system of smart home care for older adults in the community was developed, with 5 primary indices and 33 secondary indices. The weights of the 5 stair indices from high to low were smart emergency assistance 0.332, smart meal assistance 0.272, smart medical assistance 0.229, smart cleaning assistance 0.110 and smart amusement assistance 0.057.

**Conclusion:**

The results from the weight allocation revealed smart emergency assistance, smart meal assistance, and smart medical care assistance were the most important and crucial aspects of community-based smart home care. The study also suggested that “timeliness”, “reliability”, and “ease of use” should be given more attention. It is recommended to use this index system as a regulatory benchmark to guide the government bodies, senior care enterprises and communities to take measures to enhance the quality.

**Supplementary Information:**

The online version contains supplementary material available at 10.1186/s12889-023-15262-1.

## Background

Populations are ageing at an increasing rate in many nations around the world, as fertility rates decline and life expectancy rises [1]. China’s population of adults aged 65 and older in China had reached 190 million by the end of 2020, accounting for 13.5% of the total population [2]. More recently, due to the COVID-19 pandemic, prevention and control measures such as isolation, are forcing senior care services to seek new solutions by information technology and smart products. Therefore, there is an urgent need to transform and upgrade the traditional family care services. Smart home care, also known as the “intelligent senior care system” and “fully intelligent senior care system” [3], refers to the Internet of Things (IoT), computing techniques, artificial intelligence (AI), automation and intelligent systems, which are increasingly used to monitor daily conditions and support senior care activities at home [4]. These technologies are available for sensing, predicting, reminding and responding to the elderly community’s daily requirements, assisting them in a timely and socially correct way [5]. Thus, smart home care can augment existing aging resources and support the older adults to age in place to achieve ageing actively for China and even the world.

It is imperative to develop a scientific index system for evaluating the quality of smart home care, providing a reference for the government bodies, senior care enterprises and communities to take measures to enhance the quality and effectiveness of smart home care. The Chinese government developed a “Medium and Long-term Plan for actively coping with population ageing” in 2019, proposing to strengthen technological innovation capacity in response to population ageing [6]. However, smart senior care also face some challenges, such as low utilization rate caused by the digital divide, structural mismatch [7], low quality [8] and even the dilemma of having a platform but without services [9] despite of achieving initial results in recent years. To address such challenges, it is of essential importance to systematically evaluate the smart senior care.

Generally，there is a lack of panoramic and comparative analyses of smart senior care. Various studies have been conducted on the feasibility and effectiveness of smart homes or a specific technology in foreign countries. Previous studies have shown that smart home or assistive technology plays an important role in promoting the physical and mental health of the elderly individuals. They assist the elderly to live independently and support social participation, improve the quality of life of the elderly [10], and alleviate the burden on family caregivers and society [11]. For example, Cavallo, F [12] pointed out that robotic services permit older people to remain in their homes and facilitate their independent living. Additionally, Andres [13] through bibliometric network analysis, found that various technologies are beneficial for health regulation, emotion recognition, mobility, localization and fall detection. Similarly, many studies in China have shown smart products and technologies to some extent enhance the satisfaction of elderly services and improves the quality of smart elderly services [13, 14]. He Ni [15] also pointed out that the application of artificial intelligence products makes a significant contribution to the quality of senior care services in general, but varies from different products. However, only very few studies have focused on developing a quality index system of smart care in China. For instance, Geng Z et al. [16] constructed a three-level evaluation index system of smart senior care by applying the Delphi and AHP methods. Therefore, how to systematically and scientifically evaluate the quality of community-based smart home care services remain needs to be further explored.

An effective evaluation index system should reflect the elements of smart care, and should be in keeping with the prevailing situation of smart care in the community. Among many service quality assessment methods, the SERVQUAL model is the most widely used and authoritative service quality assessment tool, and the model is well-respected [17]. However, some researchers have questioned the industrial applicability of the model [17, 18]. Likewise, as some scholars have pointed out, the framework must be adapted and supplemented when necessary to suit the specific needs of certain organizations [19, 20]. As the model continues to develop and mature, the industry’s unique characteristics are gradually incorporated into the model in China, and the SERVQUAL model is thus constantly revised.

The present paper attempts to fills the gaps identified in the above literature. Different from the form of care services for seniors in institutions or nursing homes, this paper focuses on the establishment of an index system for evaluating the quality of smart home care services for older adults in the community. To construct such an index system, using the cases of Guangzhou and Shenzhen, we undertook a literature review, conducted semi-structured field interviews and utilized the Delphi method and the analytical hierarchy process (AHP) method.

## Methods

**Fig. 1:**
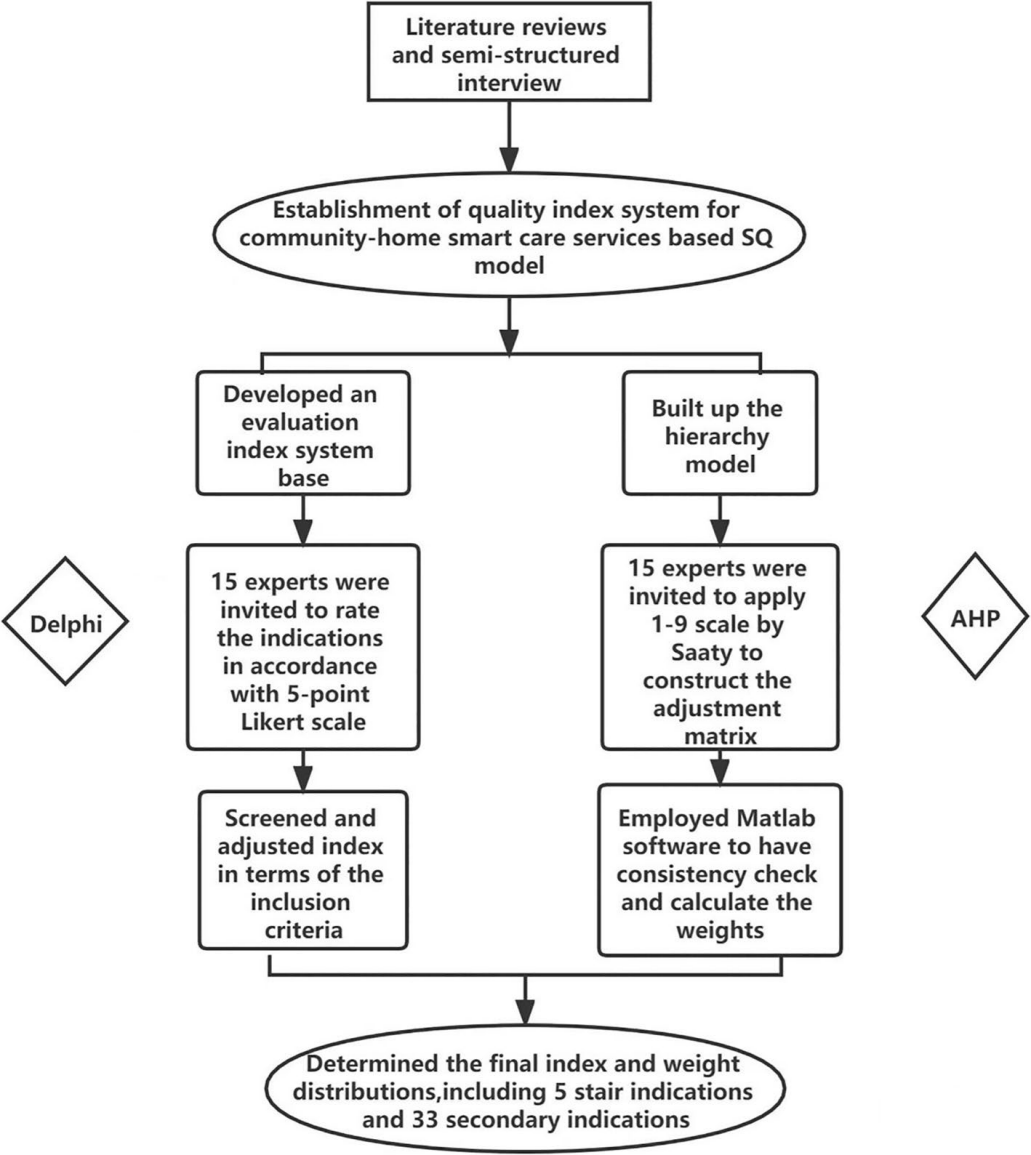
Process of determining the index and weights

On the basis of the service quality model, this study was designed as a combination of qualitative and quantitative analyses，applying the Delphi and Analytic Hierarchy Process (AHP) methods to construct the index system of smart home care in the community and obtain the weights. These were based on literature research and field interviews in Guangzhou and Shenzhen pilot districts. The flowchart depicting the process of index construction and weight determination is shown in Fig. 1.

### Define SERVQUAL dimensions

The current study focuses on the unique characteristics of “wisdom” of smart elderly care to construct evaluation indexes for the quality of smart home care for older adults in the community. To ensure that the complexity and multi-dimensionality of the service quality of smart senior care are correctly reflected, this study redefined the service quality model for the five categories of smart elderly care. Similar to the scale developed by Bo Y [21], we created five new dimensions, including tangibility, reliability, timeliness, empathy and ease of use. The study innovatively adds the “ease of use” dimension, which fully reflects the “elderly-centred” approach and take into account the reality of technology anxiety among the elderly in the context of smart care. Several studies have found that one of the primary reasons for limited use of smart senior care is the lack of simplicity with which smart equipment and systems can be learned and operated [20, 22]. Moreover, privacy and security are also key factors when it comes to accepting smart senior care products and services [23–25]. Therefore, we proposed incorporating the aspect of privacy and security into the “reliability” dimension. Additionally, we redefined the dimension of “responsiveness” into “timeliness” to stress both responsiveness to elderly users’ needs and timeliness of the service delivery. Herein, the five dimensions and definitions of the service quality model in this study are as follows:

- ◆ Tangibility: refers to the image engineering of senior care services, such as the degree to which smart devices and platforms are available.
- ◆ Reliability: refers to the provision of safe, secure, effective and satisfactory senior care services for the elderly, and also focuses on the privacy and security of elderly individuals.
- ◆ Timeliness: refers to the platform providing services for the elderly on the time agreed, upon and responding to the emergency requirements quickly.
- ◆ Empathy: refers to the platform providing targeted and customized services and differentiated pricing according to the actual situation of elderly individuals.
- ◆ Ease of use: refers to the simple interface design of the smart senior care app, system and equipment as well as easy learning, operation and time savings.

### Constructing index system

#### Semi-structured field interview

The field interviews, a total of 8 heads and managers of smart home platform in pilot districts in Guangzhou and Shenzhen cites, were conducted in 2022. Field interviews, lasting 45-90 min, were made to each participant. To begin with, the surveyor obtained informed consent from each participating respondent. In the first part of the field interview, basic information on the participants was gathered, including name, age, education level, working experiences. Moreover, each participant was asked about the current demand and supply of smart home care services in the local community, existing problems and suggestions. Participants were included in the study if they worked in the field of elderly care ≥3 years, provided written informed consent and volunteered to actively participate in the study. On the basis of literature review, the main service contents and categories of community-based smart home care services are initially refined. Then, two team members used thematic analysis to try to code the interview transcript and unified the contents and meanings of the codes mentioned in the text together. Finally, we initially developed a quality evaluation index system draft.

### Adjustment of the index system through Delphi method

The Delphi method was then used to screen evaluation indicators. The results of related studies have concluded that the number of consultation experts should be 15 ~ 50 [26]. Accordingly, a total of 15 experts were invited from universities, research institutes, senior care units, associations and hospitals to participate in the construction of the quality evaluation index system of smart home care services for older adults in the community. The selected experts not only have profound knowledge of the health professions, but also have experience in the elderly care industry, thus assuring the reliability of the consultation findings.

The inquiry questionnaire for this study was self-designed and consisted of four parts: basic information of the experts, indicator evaluation questionnaire in accordance with the initial index system, experts’ opinion on the degree of authority, and experts’ judgement basis. Each item was rated on a Likert 5-point rating scale: not important, somewhat important, general, quite important, and very important [27]. The expert group rated on a scale of 1–5 according to its importance and provided suggestions. Microsoft Excel 2010 software was used to create a database, and SPSS software was used to conduct the analysis. The full score rate, importance mean, standard deviation, and coefficient of variation of each indicator were calculated. The criteria for the change of the indexes in the experts’ correspondence were: any one of the perfect score rates < 30%, mean < 4.0, and coefficient of variation > 0.4 in the 1st round; any two of the perfect score rates < 30%, mean < 4.0, and coefficient of variation > 0.25 in the 2nd round were satisfied at the same time. In addition, if there were two or more experts with the same opinion or suggestion, the opinion and suggestion made by one expert were adopted and eventually adjusted and amended after group discussion by combining the index structure, content, and expert background.

### Weight assessment of the quality evaluation index

The index weight is a vital parameter of the quality evaluation index of intelligent elderly care. It reflects the relative importance of the index and has a direct impact on whether the result of the evaluation system is scientifically accurate. The service quality of smart home care for older adults in the community is a complex, multidimensional concept that is perceived in many ways. AHP is an effective method to convert experts’ subjective attitudes of different evaluation factors into quantitative weights, which is suitable for problems that need to be decomposed in a complex way. Hence, we employed the analytic hierarchy process to determine the weights of the index system in this study. The calculation process of the AHP method was illustrated as Fig. 1.

#### Establishment of the hierarchical structure model

**Fig. 2:**
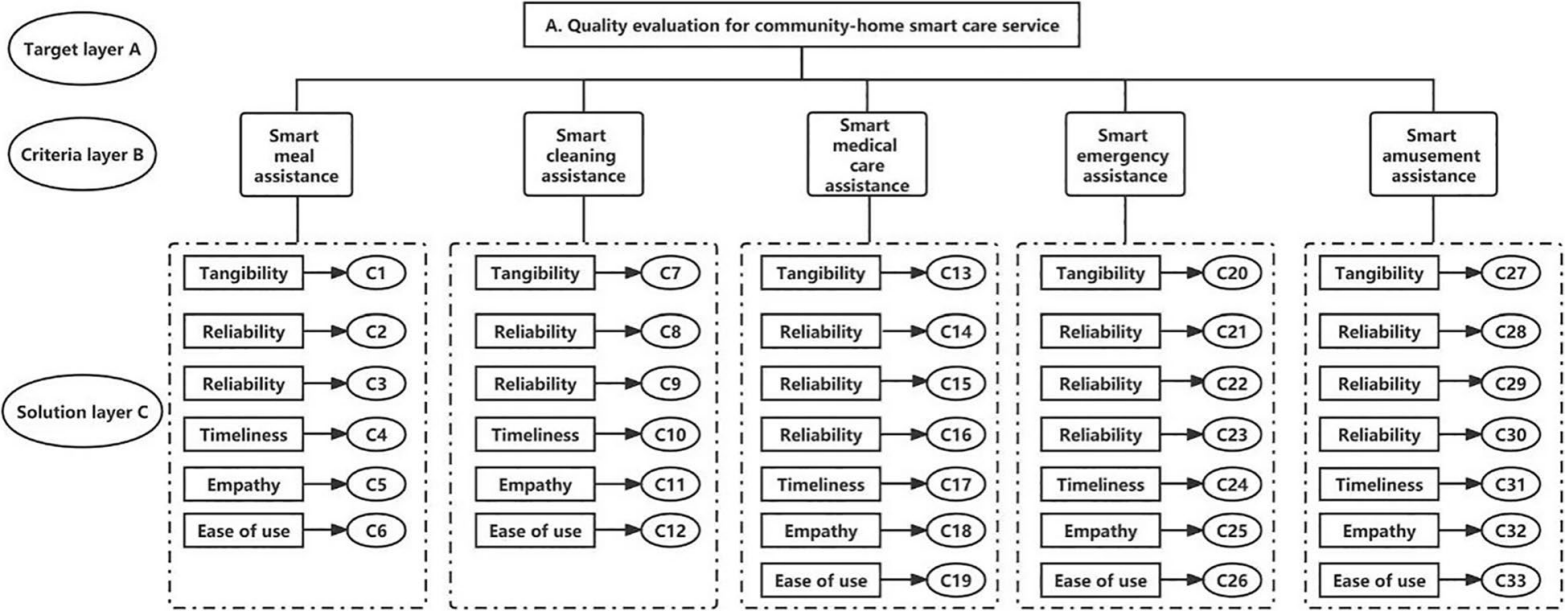
Service quality evaluation index system of community-based smart home care

The hierarchical structure model of community-based smart home care for seniors was comprised of three levels, including the target layer (the highest level), criterion layer (middle level) and solution layer (the lowest level). The target layer A was set as the overall quality of the smart home care service system. The criterion layers B1, B2, B3, B4 and B5 represented 5 different service contents, including smart meal assistance, smart cleaning assistance, smart medical care assistance, smart emergency assistance and smart amusement assistance respectively. The solution layer C was formed based on the service quality model, and contained the items C1-C33. The complete hierarchical structure for the quality evaluation is shown in Fig. 2.

#### Building the expert pair comparison matrix

The construction of a judgement matrix is an essential part of hierarchical analysis. It can help us determine whether the logic of experts is consistent. If the established matrix is not reasonable, the final weighting results obtained will be unscientific. We invited 15 experts to use the 1–9 scale method [28] to construct the two-to-two comparison judgement matrix because it is much clearer and easier for experts to determine the importance value of each indicator. In the present paper, 5 criterion layer indices and 33 solution layer indices were divided into a total of 6 matrices. The fundamental AHP numerical scale is listed in Table 1.

**Table 1:** Fundamental AHP numerical scale for verbal judgement

Numerical Value (a_**ij**_)	Verbal judgement	Interpretation
1	Equal importance	The two contribute the same to the goal.
3	Moderate importance	One is moderately more important than the other.
5	Strong importance	One is strongly more important than the other.
7	Very strong importance	One is very strongly more important than the other.
9	Extreme importance	One is extremely more important than the other.
2,4,6,8	Intermediate values of two adjacent levels	Adopted for compromising.
The Reciprocal of the above value	Inverse comparison	If the element i is compared with the element j to get the judgement a_ij_, then the judgement of comparing factor j with i a_ji_ = 1 /a_ij_

#### Weight coefficient calculation and consistency test

MATLAB2017b software was applied to calculate the weight value and perform a consistency check of all the established judgement matrices. Note that at most 10 % of this RI is suggested by Saaty [29] as the tolerable value for CI of the pairwise comparative judgement matrix, i.e., CR < 0.10. Thus, if the consistency inductor CI is less than 0.10, the comparison matrix will be strongly consistent, and the weight value will meet logical consistency. When the consistency inductor CI is not satisfied, that is, CI > 0.10, the Hadamard model will be adopted to adjust the judgement matrix [30] and when the correlation coefficient is closer to 1, the better the effect is. In this fashion, the final consistency of all the indices at all levels was verified by a consistency test, with CR < 0.1.

For the judgement matrix constructed by each expert, the weights were calculated separately by MATLAB software. Then the final importance of each index was obtained by geometric averaging of the results of 15 experts. Specifically, on the basis of the experts’ two-by-two comparison of the indicators with reference to Saaty’s fundamental 1 ~ 9 scaling method and the construction of the judgement matrix, we proceeded as follows. First, the geometric mean of each row of the judgement matrix was calculated using the product square root method, in which “a_ij_” denotes the element of the i-th row and j-th column of the primitive judgement matrix, and “n” denotes the quantity of indicators. The geometric mean of each line is normalized to obtain the feature vector, which is the weight coefficient of each indicator. Next, the consistency test of the judgement matrix is conducted: by calculating the maximum eigenvalue of the judgement matrix as: $\uplambda \textrm{max}=\frac{1}{\textrm{n}}\sum_{i=1}^n\frac{\Big(\sum_{j=1}^n{a}_{ij}{w}_{j\Big)}}{w_i}$; calculating the consistency index (CI) with the following formula: $\textrm{CI}=\frac{\uplambda \textrm{max}-\textrm{n}}{\textrm{n}-1}$; calculating the consistency ratio (CR) as: $\textrm{CR}=\frac{\textrm{CI}}{\textrm{RI}}$. RI is the average random consistency index, and its value depends on “n” and is the average consistency index of randomly generated reciprocal matrices.

Taking the elements of target layer A indicators (B1, B2, B3, B4,B5,B6) as an example, expert 1 assigned the degree of influence a_ij_ of each indicator on “A” indicators, and the judgement matrix constructed is shown in Table 1. MATLAB software was applied to calculate and check the consistency according to the above steps leading to λmax = 5.193, CI = 0.048, CR = 0.043. Since CR < 0.10, the matrix passes the consistency test. Therefore, analysis of this matrix can be performed directly. Running hierarchical analysis in this way yielded the weights of the five first-level indicators as presented in Table 2.

**Table 2:** Judgement matrix

Indicators	B1	B2	B3	B4	B5	weights
B1	1	2	1/4	1/5	3	0.099
B2	1/2	1	1/7	1/6	1	0.051
B3	4	7	1	3	9	0.495
B4	5	6	1/3	1	8	0.312
B5	1/3	1	1/9	1/8	1	0.043

## Results

### Quality index of smart home care services for older adults in the community

Five main themes were identified in the analysis of the interview data by thematic analysis: (1) catering services, (2) domestic services, (3) medical care services, (4) emergency services and (5) amusement services. As a result, we have concluded five major types of smart services, including smart meal assistance, smart cleaning assistance, smart medical care assistance, smart emergency assistance and smart amusement assistance. The specific service item classifications are shown in Table 3.

**Table 3:** Service item classifications of smart home care in the community

Service content	Service item
Smart meal assistance	Centralized card swipe, face swipe dining
	Telephone or online reservation for home cooking
Smart cleaning assistance	Personal cleaning and hygiene
	House cleaning
	Door-to-door laundry
	Electronic health data
Smart medical care assistance	Equipped with intelligent monitoring equipment
	Online consultation, hotline, online appointment services
	On-site chronic health management, physical care and rehabilitation exercises
	Equipped with intelligent emergency equipment
Smart emergency assistance	Emergency Assistance Services
	Equipped with intelligent voice chat and companion robot devices
	Hotline or online appointment for chat, counselling, get-togethers
Smart amusement assistance	Conducting online activities
	Accompanying outings

Two rounds of expert consultations were conducted in this study. The basic information concerning the experts is shown in Table 4. The participation rate of experts in both rounds was 100%, indicating a high motivation of experts to participate. The effective rate of both rounds of questionnaires was 100%. The authority coefficients of the experts were 0.883 and 0.867 respectively, both of which exceeded 0.7, showing that the experts had a high degree of authority [31]. Thus, the results are reliable and persuasive. In the first round of consultation, the mean importance of each index item was between 4.13–4.87, and the overall coefficient of variation was between 0.072–0.202. In this round, we revised four indicators based on expert opinion and group discussion. In the second round, the mean importance of each index item was between 3.87–4.87, and the overall coefficient of variation was between 0.072–0.236. In this round, additionally, 3 items with a mean importance < 4 were retained after discussion.

**Table 4:** Demographic information of the experts (*n* = 15)

Basic information	N	Composition ratio (%)
**Gender**		
Male	4	26.7
Female	11	73.3
**Age**		
≤ 40	5	33.3
41 ~ 50	6	40
51 ~ 60	4	26.7
>60	0	0
**Title**		
None	2	13.3
Lecturer	1	6.7
Associate Professor	6	40
Deputy Chief Nurse	2	13.3
Professor	4	26.7
**Education level**		
Bachelor degree or below	3	20
Master’s Degree	5	33.3
PhD degree	7	46.7
**Professional orientation**		
Social Security	5	33.3
Health Management	6	40
Senior Care	2	13.3
Nursing care	2	13.3
**Professional years**		
≤ 10	7	46.7
11 ~ 15	4	26.7
16 ~ 20	3	20
>20	1	6.7
**Workplace**		
Universities	10	66.7
Research Institutes	1	6.7
Hospitals	1	6.7
Senior Service Centers	2	13.3
Associations	1	6.7

**Fig. 3:**
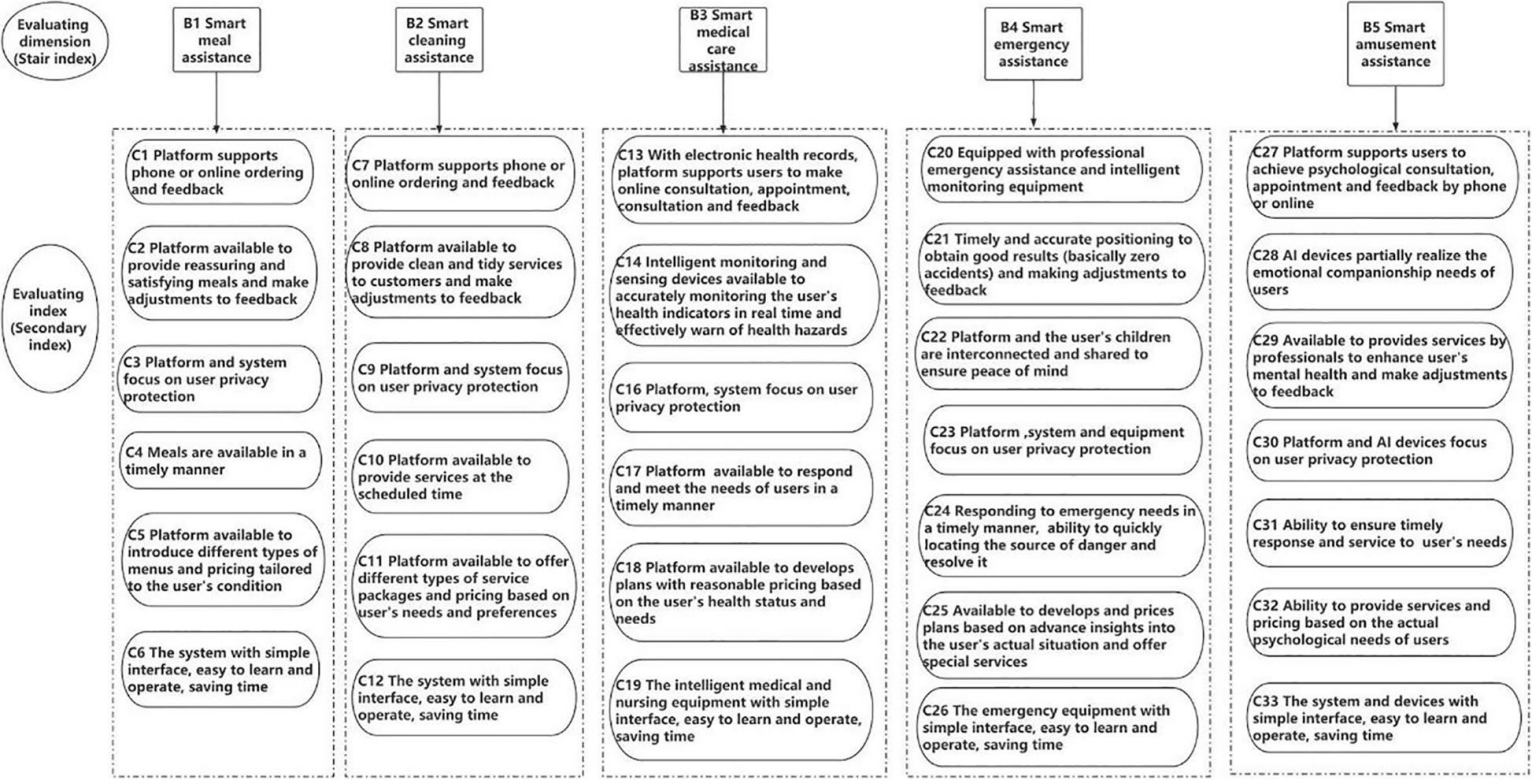
The quality index system for community-based smart home care services

After the two rounds of expert consultation, the coordination coefficients of the indices increased from 0.137 and 0.233, and the significance test of the coordination coefficients was *P*<0.01, which indicated that the experts’ opinions converged. Thus, all indices were included in the evaluation system based on exclusion criteria. The confirmed service quality evaluation index system included five criterion layer index and 33 indicator layer index. All these results are shown in Table 5 and Fig. 3.

**Table 5:** Delphi expert authority coefficient, degree of opinion concentration and opinion coordination

Rounds	Cs	Ca	Cr	Μ (min- max)	***SD*** (min- max)	Full score ratio (min-max)	W	χ^**2**^	***P***-value
Round 1	0.867	0.9	0.883	4.13–4.87	0.35–0.941	0.33–0.867	0.137	76.124	<0.01
Round 2	0.840	0.893	0.867	3.87–4.87	0.35–0.915	0.20–0.867	0.233	129.060	<0.01

*Cs* Judgement Coefficient, *Ca* Familiarity Coefficient, *Cr* Expert Authority Coefficient, *Μ* Mean, *SD* Standard Deviation, *W* Kendall coordination coefficient

### Weight distribution of the quality evaluation index

The weight coefficient of the whole quality evaluation index was built at all levels employing the analytic hierarchy process method. The weights of the 5 primary indices, from high to low were smart emergency assistance (0.332), smart meal assistance (0.272), smart medical care assistance (0.229), smart cleaning assistance (0.110) and smart amusement assistance (0.057). The weights of each level index are listed in Table 6.

**Table 6:** Community-based smart home care services system weight calculation results

Criterion layer weight	Solution layer weight	Comprehensive weight
B1 0.272	C1 0.152	0.041
	C2 0.172	0.047
	C3 0.186	0.051
	C4 0.179	0.049
	C5 0.138	0.038
	C6 0.182	0.050
B2 0.110	C7 0.153	0.017
	C8 0.216	0.024
	C9 0.144	0.016
	C10 0.190	0.021
	C11 0.116	0.013
	C12 0.180	0.020
B3 0.229	C13 0.152	0.035
	C14 0.163	0.037
	C15 0.187	0.043
	C16 0.095	0.022
	C17 0.157	0.036
	C18 0.101	0.023
	C19 0.153	0.035
B4 0.332	C20 0.139	0.046
	C21 0.185	0.061
	C22 0.144	0.048
	C23 0.103	0.034
	C24 0.245	0.081
	C25 0.117	0.039
	C26 0.138	0.046
B5 0.057	C27 0.146	0.008
	C28 0.121	0.007
	C29 0.200	0.011
	C30 0.131	0.007
	C31 0.160	0.009
	C32 0.116	0.007
	C33 0.126	0.007

## Discussion

Smart care is proposed based on screening and addressing the needs of elderly individuals, identifying and resolving the risks of the elderly and achieving the realistic goal of meeting their personalized and multilevel service needs and demands [7]. Coinciding with the intersection of the silver-hair wave and the digital wave, intelligent care is currently one of the most critical tasks in most countries and areas. With the purpose of realizing sustainable development of smart care, the key point is to conduct scientific evaluation norms and guidelines. However, smart care services in China are still in the initial stage, and further research into how to systematically and scientifically evaluate the quality of smart home care services for community-dwelling older individuals is much needed. In this work, to ensure that older adults ageing in place receive high-quality senior care services and to promote the full flowering of smart senior care, we have drawn on the SERVQUAL model and combined qualitative and quantitative analyses using the Delphi and AHP methods to construct the index system for smart senior care and obtain the weights.

### The characteristics of quality index system for smart home care

In sum, the quality evaluation index system for smart home care services for older adults in the community identified in this study has the following characteristics. First, the index system better reflects the connotation of smart care for the elderly, and highlights the characteristics and advantages of “wisdom” in the process of aging service provision. Secondly, the evaluation indices are more optimized and have stronger realism. In the process of index development, we also took into account the “fear of technology” among the elderly and their concern about the leakage of personal privacy in the process of smart senior care development in the era of big data. Rooted in China’s national conditions, the final secondary indicators are directly measurable indicators based on the actual situation in Guangzhou and Shenzhen, which can be used to intuitively measure the evaluation of elderly users on the quality of smart senior care services.

### Result of index weight analysis

From the criteria level, the weighted value of the five established first-level indices is shown in Table 6: smart emergency assistance (0.332), smart meal assistance (0.272) and smart care assistance (0.229). This, indicates that these three indices are the key parts used to measure the quality of community smart home care, whereas the weight values of smart cleaning assistance and smart amusement assistance are relatively lower, namely, 0.110 and 0.057 respectively. The results from the current study suggest that when constructing the smart senior care service system, the development, design, popularization and service quality improvement of equipment related to emergency assistance, meal assistance and medical care assistance must be prioritized. Actually, through wearable devices and intelligent monitoring service devices, intelligent emergency assistance and intelligent medical assistance can realize the functions of positioning, monitoring and assisting elderly individuals. This can help the elderly achieve independence and effectively improve the quality of intelligent elderly care and help the development of smart elderly care. Smart assistance for emergency and smart assistance for medical care was affirmed by foreign researchers earlier. They have also advocated the expansion of the coverage of information technology in home care services [32] and fully exploit smart devices and technologies such as remote intelligent control to respond quickly to remote calls for assistance from elderly individuals. This will lower the cost of medical care integration, and alleviate the shortage of care services [33]. Similarly, a study has also proposed that at present, the fields with a high embedding degree of Internet technology largely focus on delivering meals, life care and medical services. However, due to the inherent deficiency of Internet technology in providing spiritual comfort [34], the embedding degree of Internet technology is limited, and it is difficult to fully rely on relevant devices or terminals. They can only play a supplementary role [35]. It is noteworthy that, as the development of smart elderly related industries is still in the preliminary stage, the content of smart elderly services still has limitations and lacks uniformity. In the future, with the continued growth of smart senior care, the weight of smart amusement assistance has more room to rise, which is also in harmony with the idea of some foreign scholars. For instance, Godfrey [36] suggested that science and technology should be integrated into the traditional elderly care model to innovate the social interaction of the elderly through information technology. Similarly, Khaksar [10] argued that nursing homes should use social robots to help older adults overcome social barriers and innovate smart senior care services from theory to practice.

From the program level, the indicators with higher weight values are mostly focused on the three dimensions of timeliness, reliability and ease of use. In other words, efforts must be made in the following areas during the construction of smart home care services for older adults in the community: guarantee timely response to user needs and timely provision of services; secure satisfactory services provided by professionals [37], enhance the ability of the platform to make adjustments based on user feedback and to stress confidentiality of user information [24, 38, 39]; ensure that the design of smart devices and systems is age-friendly and eliminate the digital divide as much as possible [37]. Additionally, the finding from the weight values of the secondary index, it shows that the weights of the indicators under the “empathy” dimension is generally the lowest. The major reason for this is that the Internet has not yet been deeply integrated into the senior care industry in China. This, makes it difficult to provide personalized and customized services of different types and pricing in accordance with the situation, needs and preferences of elderly users.

The combination weights are able to reflect the importance of the secondary indicators for all service components. In the combination weights, there are 15 indicators that exceed the average weights. The high indicator weights are largely distributed under the three dimensions of timeliness, reliability and ease of use of each service content. This is basically consistent with the results of the weights of the primary and secondary indicators, also implying that these three dimensions have a significant status and role in the evaluation of smart home care services for older adults in the community. Yang Bo in China [21] first tried to construct a scale for the quality of smart home care services, and concluded that the five service quality dimensions in descending order of weight were: reliability>empathy>timeliness>ease of use>tangibility. Our study concludes that timeliness, reliability, and ease of use are more important, which is consistent with previous findings to some extent [21]. However, studies have produced a conflicting result in regard to the importance of “empathy” [21]. The inconsistent findings could be caused by the heterogeneity of perspectives and cultural backgrounds of those interviewed and the consultation participants. Therefore, further studies are needed to confirm the importance of the different dimensions.

### Strength and limitation

This study has established a system for evaluating the service quality of intelligent home care services for seniors in the community. To begin with, this index system can be used to evaluate the current situation of smart home senior care service quality, summarize the existing problems, explore the key factors and effective paths to achieve high quality. This index system, furthermore, may also be used as a regulatory benchmark to guide the government, senior care enterprises and communities to take measures to advance the quality. However, there are still some shortcomings in the study. First, the reliability, differentiation and representativeness of the indicators in the index system need to be further tested by specific applications. Accordingly, in the future, we will use this index system to conduct field research among elderly users, so that the evaluation index system can be continuously improved. Furthermore, since the constructed evaluation index system is based on the specific practices in Guangdong Province, the external probability validity of the service evaluation index system constructed in this study may have certain limitations. Thus, further in-depth research is needed to expand its empirical scope. Next, we will use this index system to conduct field research among the users of smart home care services, so that the evaluation index system can be continuously improved.

## Conclusion

Smart home care for the elderly in the community is the future of the silver ageing industry. It is imperative to evaluate the service quality based on the actual practice status, and emphasize the “ people-centeredness “ nature of the service, to achieve the goal of high-quality development. In summary, this study has contributed to the development of indicators for evaluating the quality of smart home care for China’s community-dwelling older adults. In compliance with the applicable conditions in the pilot cities in Guangdong Province, a quantitative quality evaluation index system was constructed. In the present study, the outcomes revealed that more resources should be invested into smart emergency assistance, smart meal assistance and smart medical assistance, which have higher weights among the five smart elderly service items. In addition, the weight distribution of the secondary evaluation index have demonstrated it is of essential importance to prioritize the timeliness, reliability, and ease of use of smart senior care services, thereby facilitating the quality of smart senior care services. These findings lay a good foundation for further research on the quality evaluation of smart care for community-dwelling elderly individuals. Finally, in the era of intelligence, the role of information technology and intelligent products in meeting the spiritual needs of the elderly is still severely limited. Hence, more humanistic care should be given to the older adults to raise the temperature of elderly services.

## Supplementary Information

**Additional file 1.** Judgement Matrix of smart medical care assistance.

**Additional file 2.** Judgement Matrix of smart meal assistance.

**Additional file 3.** Judgement Matrix of smart emergency assistance.

**Additional file 4.** Judgement Matrix of smart cleaning assistance.

**Additional file 5.** Judgement Matrix of smart amusement assistance.

**Additional file 6.** Importance rating of index—second round.

**Additional file 7.** Importance rating of index—first round.

**Additional file 8.** 2.Expert authority.

**Additional file 9.** 1.Demographic information of the experts.

## Data Availability

Data sharing is available upon reasonable request, during the present study. Readers can contact Li Wang (trmd_2002@163.com) to submit raw data.

## Abbreviations

AI: Artificial intelligence
IoTs: the Internet of Things
SQ Model: SERVQUAL Model, Service Quality model
AHP: Analytic Hierarchy Process
C_r_: Expert authority coefficient
C_s_: Judgement coefficient
C_a_: Familiarity coefficient
M: Mean
*SD*: Standard Deviation
